# Effect of Activation Modes on the Property Characterization of Crumb Rubber Powder from Waste Tires and Performance Analysis of Activated Rubber-Modified Asphalt Binder

**DOI:** 10.3390/polym14122490

**Published:** 2022-06-19

**Authors:** Honggang Zhang, Yangpeng Zhang, Jie Chen, Wenchang Liu, Wensheng Wang

**Affiliations:** 1 School of Traffic and Transportation Engineering, Changsha University of Science and Technology, Changsha 410114, China; 2 Guangxi Transportation Science and Technology Group Co., Ltd., Nanning 530007, China; cj_engineering@163.com; 3 Guangxi Key Lab of Road Structure and Materials, Nanning 530007, China; 4 School of Materials Science and Engineering, Chang’an University, Xi’an 710064, China; lwc_engineering@163.com; 5 College of Transportation, Jilin University, Changchun 130025, China; wangws@jlu.edu.cn

**Keywords:** waste crumb rubber, activation mode, rubber characterization, modified asphalt binder

## Abstract

The rubber molecular chain in waste vulcanized tire rubber will be crosslinked to form a network structure that would be difficult to degrade in asphalt. Crumb rubber treated by desulfurization activation could form active groups on the surface by interrupting the crosslinking bond to improve the compatibility between crumb rubber powder and asphalt. To explore the influence of activation modes on crumb rubber powder and the corresponding rubber-modified asphalt binder, crumb rubber powder was firstly activated through three commonly used activation methods and asphalt binder samples modified by activated crumb rubber powder were also prepared. The basic properties of activated crumb rubber powder were characterized by infrared spectroscopy, and conventional tests were used to study the conventional physical properties of the asphalt binder. The infrared spectroscopy and elemental analysis showed that the crumb rubber powder was mainly composed of alkanes, alkenes, sulfonic acids, aromatics, and a little silica rubber and antioxidant zinc oxide, which is suitable for asphalt modification. The simple heat activation treatment method is not enough to greatly destroy the cross-linking structure of crumb rubber powder, but the “C=C” bond was destroyed more seriously. Under the action of adjuvants, the polysulfide cross-linking bond could be broken in crumb rubber powder. The heat treatment and chemical treatment could not achieve the purpose of reducing the viscosity and improving the compatibility of rubber asphalt binder through desulfurization activation. The mechanochemical treatment would help to improve the performance of crumb-rubber-powder-modified asphalt binder. The data correlation analysis based on the grey relational degree can provide a reference for the selection of activated crumb rubber powder for different application requirements in the asphalt modification procedure.

## 1. Introduction

In recent years, with the rapid development of new solid waste utilization technology in the road industry, renewable materials-modified asphalts represented by waste crumb rubber powder have been widely used in the field of civil engineering [1,2,3]. Scholars have systematically studied the modification process, road performance, and application of waste crumb-rubber-powder-modified asphalt in hot mix asphalt mixtures [4,5,6]. To further improve the utilization efficiency of waste crumb rubber and improve the road performance of crumb-rubber-modified asphalt binder, many scholars have studied the activation process of crumb rubber powder.

Due to the rubber molecular chain, it would be difficult for waste vulcanized tire rubber powder to degrade in asphalt binder [6]. Crumb rubber after desulfurization activation could form active groups on the surface by interrupting the crosslinking bond to improve the compatibility between crumb rubber powder and asphalt [7,8]. At present, the commonly used crumb rubber powder activation methods include physical activation methods, such as the microwave method and ultrasonic method [9], chemical activation methods [9], the biological method [10], etc. In addition to the above activation methods, mechanochemical methods using chemical adjuvants are also applied to the activation of crumb rubber powder [11]. Many researchers have believed that the storage stability and workability of crumb-rubber-modified asphalt binder prepared from rubber powder have been significantly improved after desulfurization activation [12,13]. Juganaru et al. observed that according to the micro morphology of activated crumb-rubber-powder-modified asphalt binder, the activated crumb rubber powder was more evenly distributed in asphalt, and the viscosity of activated crumb-rubber-powder-modified asphalt binder was significantly reduced [14]. Shatanawi et al. pointed out that the water stability and rutting resistance of activated crumb-rubber-powder-modified asphalt mixtures are better than those of ordinary crumb-rubber-modified asphalt mixtures [15]. Liu et al. used the wet process to prepare a crumb-rubber-modified asphalt binder with different activated powder contents and optimized the preparation parameters of activated crumb rubber powder based on the viscosity and softening point difference. The asphalt mixture containing 60% content of activated crumb rubber powder possesses optimal comprehensive properties in terms of high-temperature rutting resistance, cracking resistance, and moisture resistance [16]. Chen et al. studied a new treatment method of crumb rubber impact on high temperature rheological performances and found that activated crumb-rubber-powder-modified asphalt binder has better rheological performances at high temperatures, as well as better short-term aging performances [17].

The above studies showed that activated crumb rubber powder can improve some properties of crumb-rubber-modified asphalt binder, but activated crumb-rubber-powder-modified asphalt binder still has problems such as difficult construction and poor aging resistance. To solve the above problems, researchers have conducted in-depth research in the field of crumb rubber powder activation and regeneration and achieved fruitful results [18,19,20,21]. The activation of crumb rubber powder is a method of pretreatment before asphalt modification to improve the physicochemical properties of crumb rubber powder. Its main purpose is to keep the core of the rubber powder particles and the “C-C” main chain from being damaged and to try to open the “S-S” bond and “C-S” bond in the crosslinking network [22]. After desulfurization and activation, the surface of crumb rubber powder becomes rougher and some rubber recovers the properties of raw rubber, its compatibility with asphalt is greatly enhanced, and problems such as high viscosity and easy segregation of crumb-rubber-modified asphalt binder are significantly improved [23,24]. Chen et al. applied a surface treatment on crumb rubbers to improve the aging resistance of the asphalt rubberized binder. After the pressure-aged vessel (PAV), the bending beam rheometer (BBR) test at −6 °C, −12 °C, and −18 °C, the linear amplitude sweep (LAS), and dynamic shear rheometer (DSR) test were used to evaluate the cracking resistance and fatigue resistance. The binders produced with medium-sized rubber had good low-temperature properties, and softer virgin asphalt generally had better cracking resistance [25]. Xie et al. introduced two activation methods to treat crumb rubber and improved the high- and low-temperature performance and decreased the softening difference by 79.8%. The interaction between rubber and asphalt binder was enhanced through the activation of rubber, which could solve its poor storage stability [26]. Chen et al. added reacted and activated rubber to improve the elastic properties and decrease the viscous features of the binders. At a strain level of 5.0%, the binder fatigue life of 35% reacted and the activated rubber was 43 times higher than that of the control binder [27]. Zhou et al. studied the physical and chemical changes before and after aging of asphalt modified by microwave activated crumb rubber. They found that microwave activation made the swelling reaction more intense, thereby delaying the conversion of the large molecular size in the rubber asphalt [28]. Wei et al. prepared activated crumb rubber using a hydrogen peroxide solution and found that with the increase in solution content, the contact surface between the particles increased, the floccules and pores of the powder increased, and the interface degree between the crumb rubber powder and the asphalt was strengthened [29].

Crumb-rubber-powder-modified asphalt binder has been proven to improve the road performance of asphalt materials. However, due to the change of physical and chemical properties before and after rubber powder activation, it is different from the original crumb-rubber-modified asphalt binder in the modification system, which will have an impact on the modification technology. Therefore, crumb rubber powder was firstly treated and activated through three commonly used activation methods, including heat treatment, chemical treatment, and mechanochemical treatment methods, respectively. Asphalt binder samples modified by activated crumb rubber powder were also prepared. The basic properties of activated crumb rubber powder were characterized by infrared spectroscopy, and conventional tests were used to study the conventional physical properties of asphalt binder to explore the influence of activation modes on crumb rubber powder and the corresponding rubber modified asphalt binder.

The objective of this work is to explore the influence of activation modes, including heat treatment, chemical treatment, and mechanochemical treatment methods, on crumb rubber powder and modified asphalt binder. In this study, the crumb rubber sample with 30 mesh was selected and activated using the three activation treatment methods. The physical and chemical properties of activated crumb rubber powder were characterized by infrared spectroscopy and SEM. Next, penetration, softening point, ductility at 5 °C, viscosity, elastic recovery, and softening point difference (Δ) tests were carried out to evaluate the high- and low-temperature performance and construction workability of modified asphalt binder. Based on the grey relational analysis, the influences of different activation modes on the conventional physical properties were further evaluated.

## 2. Materials and Methods

### 2.1. Experimental Raw Materials

The crumb rubber powder used in this study was the crumb rubber sample with 30 mesh provided by Guangxi Transportation Science and Technology Group Co., Ltd., Nanning, China, which was obtained by the normal temperature crushing method. According to the macro- and micromorphology of crumb rubber from the original appearance and scanning electron microscopy (SEM) in the previous study [11], the surface of the crumb rubber powder is rough and angular. Meanwhile, due to the crumb rubber powder being roughly sieved, it is composed of crumb rubber powder with different particle sizes. The sieved results of crumb rubber powder are shown in Table 1. Maoming 70# asphalt (Maoming, Guangdong, China) was used as the asphalt matrix, which has a penetration of 72 (0.1 mm), softening point of 46.0 °C, ductility at 15 °C more than 100 cm, and density of 1.08 g/cm^3^.

### 2.2. Activation Treatments for Crumb Rubber Powder Desulfurization

The commonly used activation methods for crumb rubber powder include the heat activation treatment method, chemical activation treatment method, and mechanochemical activation treatment method.

- Heat activation treatment method

(i) A certain amount of crumb rubber powder was put into an aluminum box, which was sealed and placed in an oven, and (ii) the samples were heat-treated at different temperatures (160 °C, 180 °C, and 200 °C) and different durations (30 min, 60 min, and 90 min), respectively. The heat-activation-treated crumb rubber powder and untreated crumb rubber powder were tested and characterized by the Fourier infrared spectrum.

- Chemical activation treatment method

The adjuvants used in this study include organometallic complexes, labeled as OMC, and organic disulfide, labeled as OD, provided by Hebei Richway Technology Co., Ltd., Shijiazhuang, China, and a self-made active agent prepared in a fume hood (deep eutectic solvents) labeled as DES. Considering the properties of the adjuvants, different heating temperatures were selected to prevent the spontaneous combustion of adjuvants.

For OMC or OD: (i) After swelling a certain amount of crumb rubber powder in toluene solution for 12 h, the adjuvant (OMC or OD, accounting for 3% of crumb rubber powder by mass) was added to the beaker. (ii) The mixture was heated to a constant temperature (70 °C) and blended at a speed of 2000 rpm for 1 h. After the mixture was cooled to room temperature, it was conducted by suction filtration. After suction filtration, the crumb rubber powder was washed with water 3~5 times. (iii) The treated crumb rubber powder sample was placed in a vacuum drying oven and dried to a constant weight at 60 °C, and activated crumb rubber powder by the chemical adjuvants OMC or OD could be obtained.

For DES: (i) A certain amount of crumb rubber powder and self-made active agent DES (10% crumb rubber powder by mass) were evenly mixed in the beaker, heated to a constant temperature (180 °C), and blended at the speed of 3000 rpm for 30 min. (ii) After cooling to the room temperature and suction filtration, the crumb rubber powder was washed with water 3~5 times. (iii) The treated crumb rubber powder sample was dried at 60 °C, and activated crumb rubber powder by DES could be obtained.

- Mechanochemical activation treatment method

**Figure 1:**
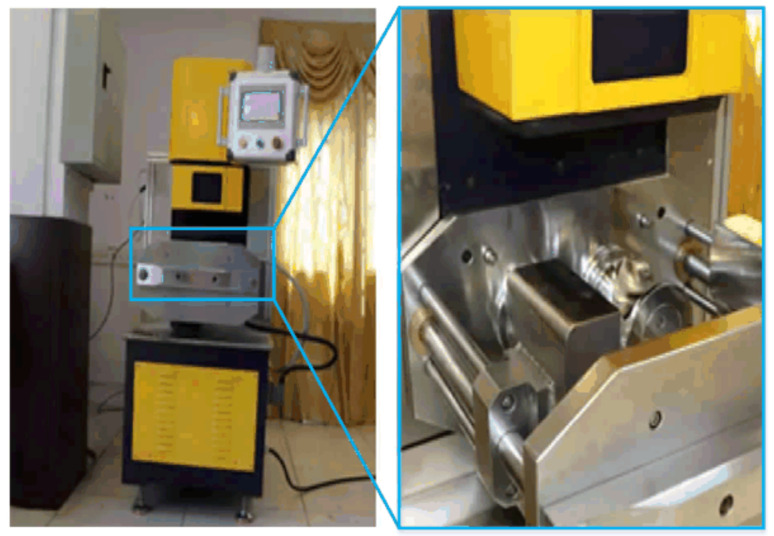
The opening and closing mixer.

The mechanochemical activation treatment method refers to applying a mechanical force to the mixture of crumb rubber powder and adjuvants to accelerate the activation between them and promote the change of structure and physicochemical properties for crumb rubber powder. The opening and closing mixer shown in Figure 1 with a capacity of 1 L was used for mixing the crumb rubber powder and adjuvants, along with a reaction vessel and mechanical force application instrument. A certain amount of crumb rubber powder and different kinds of adjuvants were put in the opening and closing mixer, and then mixed with the speed of 40 r/min at the specified temperature (120 °C) for 60 min to obtain the activated crumb rubber powder. This mechanochemical activation treatment method can effectively play the desulfurization activation role of adjuvants and extrude and crush crumb rubber powder under the action of mechanical force, which greatly increases the surface roughness and activity of crumb rubber powder.

### 2.3. Experimental Scheme and Methods

**Figure 2:**
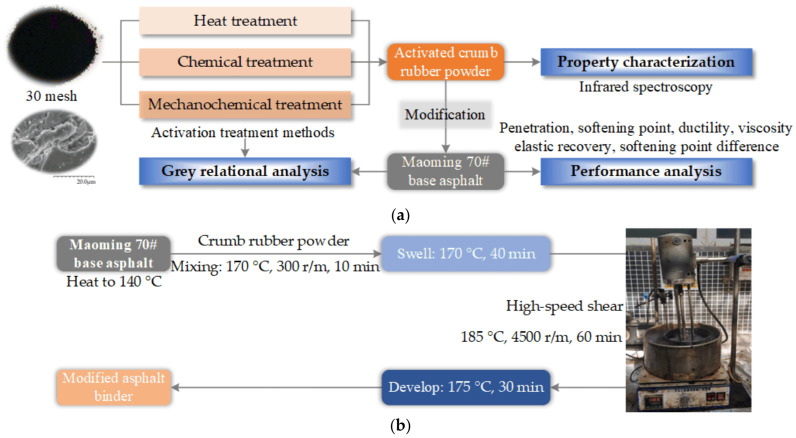
The experimental scheme and asphalt binder sample preparation: (**a**) the flow chart of this study; and (**b**) the asphalt binder sample preparation.

The purpose of this study was to guide the industrial production and application of activated crumb rubber powder. The flow chart of this study is shown in Figure 2a. Firstly, the physicochemical properties of crumb rubber powder were characterized. Then, the activated crumb rubber powder was prepared by heat activation treatment, chemical activation treatment, and mechanochemical activation treatment. The basic properties of the activated crumb rubber powder were characterized by Bruker Tensor II Fourier Infrared Spectrometer, and the effects of different activation modes on the properties of crumb rubber powder were compared and analyzed to provide a reference for subsequent asphalt tests.

Based on the properties of the activated crumb rubber powder by using different activation modes, the corresponding activated crumb-rubber-powder-modified asphalt binder was prepared as shown in Figure 2b. The basic physical properties of the activated crumb-rubber-powder-modified asphalt binder were tested, and the effects of the activation modes on the performances of the activated crumb-rubber-powder-modified asphalt binder were systematically studied. According to the Chinese standard “Standard Test Methods of Bitumen and Bituminous Mixtures for Highway Engineering” (JTG E20-2011), the penetration (T0604), softening point (T0606), ductility at 5 °C (T0605), rotational viscosity at 180 °C (T0625), elastic recovery (T0662), and softening point difference (Δ) (T0661) of the activated crumb-rubber-powder-modified asphalt binder were tested. The techniques used and test conditions were detailed in the previous study [11].

## 3. Results and Discussion

### 3.1. Analysis of the Physicochemical Properties of Crumb Rubber Powder

1. Thermogravimetric analysis

**Figure 3:**
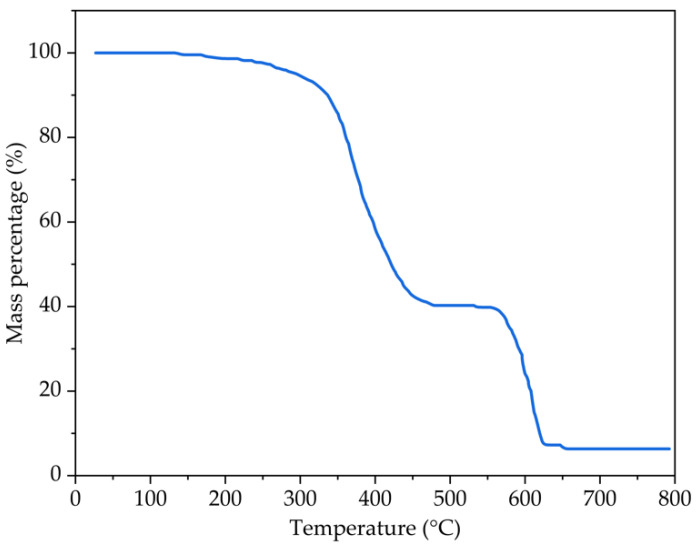
The thermogravimetric curve of crumb rubber sample with 30 mesh.

The thermogravimetric analysis on the crumb rubber powder with 30 mesh was carried out and its composition was calculated. The test results are shown in Figure 3 and Table 2.

It can be seen from Figure 3 and Table 2 that before 300 °C, there is a nitrogen atmosphere in the heating furnace. At this time, the crumb rubber powder does not burn, and the mass loss of crumb rubber powder is small, which is mainly caused by the volatilization of volatile components (VOCs) in crumb rubber powder. The VOCs in crumb rubber powder mainly include rubber raw materials, adjuvants, and water mixed or absorbed in the process of production and transportation. The organic solvents would be gradually released into the asphalt after crumb rubber powder is added into the asphalt. Most organic solvents are small molecular compounds which can improve the low-temperature performance and aging resistance of asphalt binder.

With the increase in temperature, the rubber component in crumb rubber powder begins to depolymerize, and the mass of the crumb rubber powder decreases rapidly. When the temperature rises to 550 °C, the mass of the crumb rubber powder tends to be stable. At this time, the organic components in the crumb rubber powder are completely decomposed. Rubber hydrocarbon, that is, the content of vulcanized rubber in crumb rubber powder, is the most key component in crumb rubber powder whose content directly affects the physical and mechanical properties of crumb rubber powder. Acetone extract is a variety of adjuvant in rubber products and it can be extracted with acetone under heating conditions. The rubber hydrocarbon content is obtained by subtracting the acetone extract content from the total organic matter (TOM) content.

As the temperature continues to rise and the air is introduced to fully burn the remaining components, the content of carbon black can be obtained. Carbon black in crumb rubber powder is the most important reinforcing agent in an automobile rubber tire. Carbon black is mainly used to improve the wear resistance, high-temperature resistance, and elasticity of vulcanizate. Carbon black will be gradually released in the process of rubber powder modification or asphalt modification, and the filling network structure in crumb rubber powder will be damaged, resulting in the strength reduction in the crumb rubber powder.

During rubber mixing and processing, some inorganic oxides such as zinc oxide, calcium oxide, and silicon dioxide will be added to promote the activity of rubber in the vulcanization process and improve the vulcanization efficiency. These inorganic oxides have a high melting point and will not undergo chemical changes such as decomposition at 700 °C. In addition, because of the poor high-temperature resistance of rubber materials, anti-aging agents and other substances should be added to the rubber tire manufacturing process to improve the anti-aging ability of rubber. These additives are the residues of the crumb rubber powder in the thermogravimetric analyzer at 700 °C, that is, they are the main components of ash. These additives will affect the high- and low-temperature performance and aging resistance of the asphalt binder, to a certain extent.

2. Infrared spectrum analysis

**Figure 4:**
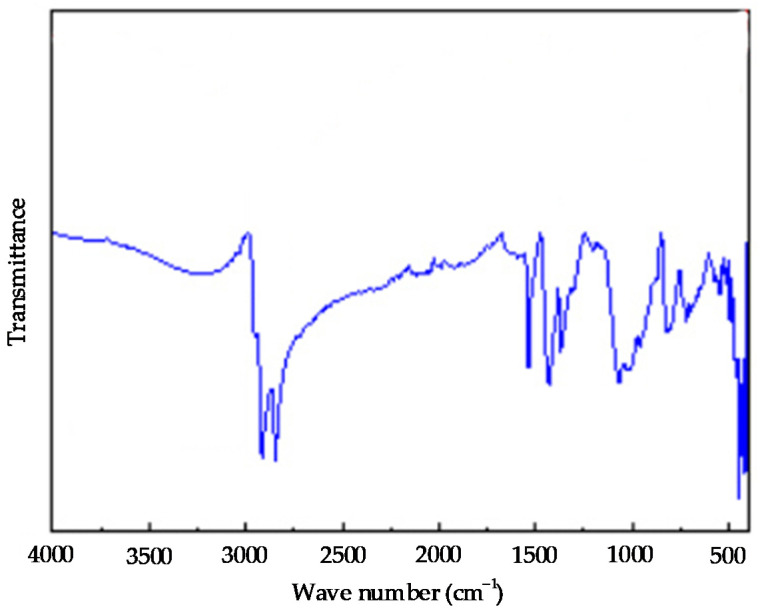
The infrared spectrum of the crumb rubber sample with 30 mesh.

The infrared spectrum of the crumb rubber powder with 30 mesh is shown in Figure 4. It can be seen from the figure that the infrared spectrum of the crumb rubber powder has strong absorption peaks at wave numbers of 2850 cm^−1^~3000 cm^−1^. It can be judged that the crumb rubber powder contains “-CH_3_, =CH_2_ and ≡CH” at the absorption peaks at wave numbers of 2952 cm^−1^, 2914 cm^−1^, and 2848 cm^−1^. The strong absorption peak at 1536 cm^−1^ in the infrared spectrum of the crumb rubber powder is caused by the stretching vibration of the “C=C” skeleton, indicating that the crumb rubber powder contains certain double bonds. The absorption peaks at wave numbers 1429 cm^−1^ and 1370 cm^−1^ are caused by the bending vibration in the “C-H” plane, and they are the bending vibration absorption peaks of the “-CH_3_“ alkane group in the crumb rubber powder. The absorption peaks near 1067 cm^−1^ and 1030 cm^−1^ in the infrared spectrum of the crumb rubber powder are caused by the stretching vibration of “C-O” in the anhydride and ether bond, or by the stretching vibration of “S-O” or “S=O”. The C-H vibration peak on the terminal alkenyl “RCH=CH_2_“ at wave number 959 cm^−1^ and the absorption peaks near the wave numbers 819 cm^−1^ and 719 cm^−1^ are caused by the bending vibration of “=C-H”. The crumb rubber powder contains functional groups mainly composed of alkanes, alkenes, sulfonic acids, aromatics, and other compounds.

3. Elemental analysis

**Figure 5:**
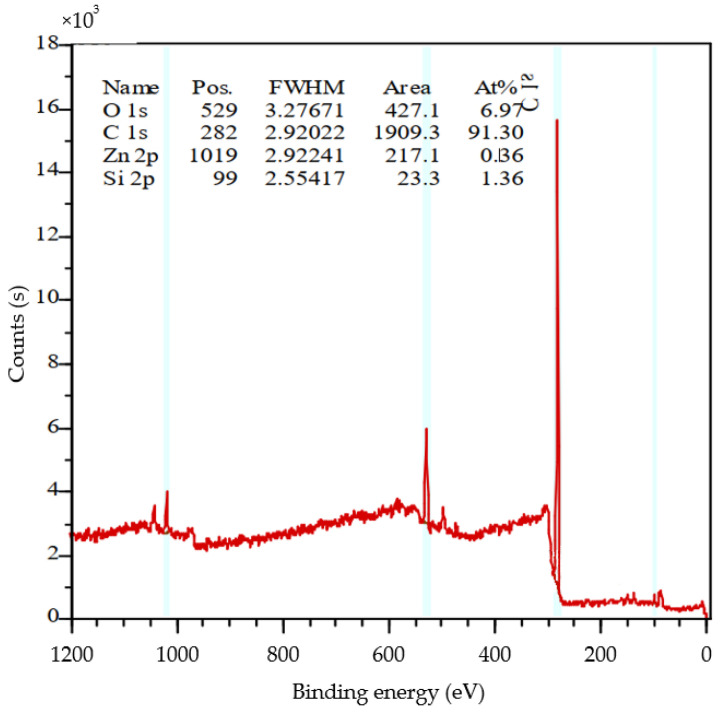
The XPS spectrum of the crumb rubber sample with 30 mesh.

The test results of the element content of the crumb rubber powder with 30 mesh are listed in Table 3, and the corresponding XPS test results are shown in Figure 5.

It can be seen from the test results that the main element of the crumb rubber powder is carbon. Through XPS full spectrum analysis, in addition to some substances known from infrared spectrum analysis, there may also be a little silica rubber and antioxidant zinc oxide in the crumb rubber powder.

### 3.2. Property Characterization of Activated Crumb Rubber Powder

#### 3.2.1. Activated Crumb Rubber Powder Prepared by the Heat Treatment Method

**Figure 6:**
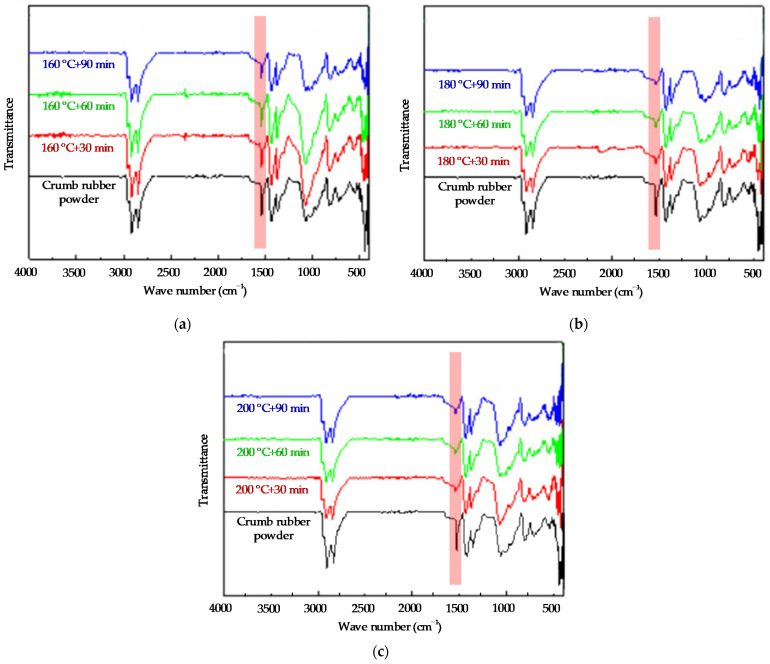
The infrared spectrum of the crumb rubber by activated by heat treatment at different durations: (**a**) 160 °C; (**b**) 180 °C; and (**c**) 200 °C.

**Figure 7:**
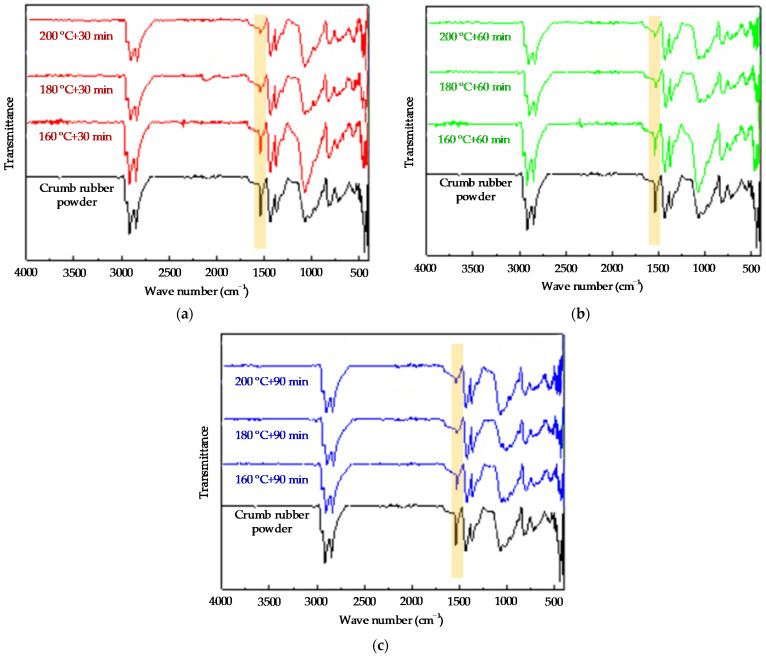
The infrared spectrum of crumb rubber by heat activation treatment at different temperatures: (**a**) 30 min; (**b**) 60 min; and (**c**) 90 min.

The infrared spectrum of the crumb rubber powder by activated by the heat activation treatment method could be obtained by Bruker Tensor II Fourier infrared spectrometer, in which the test wave number range is 500 cm^−1^~4000 cm^−1^. The infrared spectrum of the crumb rubber powder activated by the heat treatment method is shown in Figure 6 and Figure 7.

From Figure 6, it can be seen that for the crumb rubber powder prepared by the heat treatment method at the same treatment temperaturea but for different treatment durations, the curve trends of the infrared spectrum of the different activated crumb rubber powder samples are the same. However, the longer the heat treatment duration, the more serious the damage to the “C=C” bond. Not many changes are observed for the “S=O” bond at 1082 cm^−1^ and the “C=O” bond at 1710 cm^−1^. At the same time, it could be found that the damage degree of heat activation treatment to the cross-linking bonds such as the “S=O” bond and “C=O” bond is much less than that of the damage degree of the heat activation treatment to the “C=C” bond.

It can be seen from Figure 7 that when the heat treatment durations are the same but the heat treatment temperatures are different, the higher the heat treatment temperature, the more serious the damage to the “C=C” bond for the activated crumb rubber powder prepared by the heat treatment method. Thus, increasing the heat treatment temperature does not achieve the effect of promoting activation, but the “C=C” main chain in the crumb rubber powder has been destroyed. To summarize, it can be seen that the simple heat activation treatment method is not enough to greatly destroy the cross-linking structure of the crumb rubber powder and can not effectively interrupt the “C-S” and “S-S” bonds.

#### 3.2.2. Activated Crumb Rubber Powder Prepared by the Chemical Treatment Method

**Figure 8:**
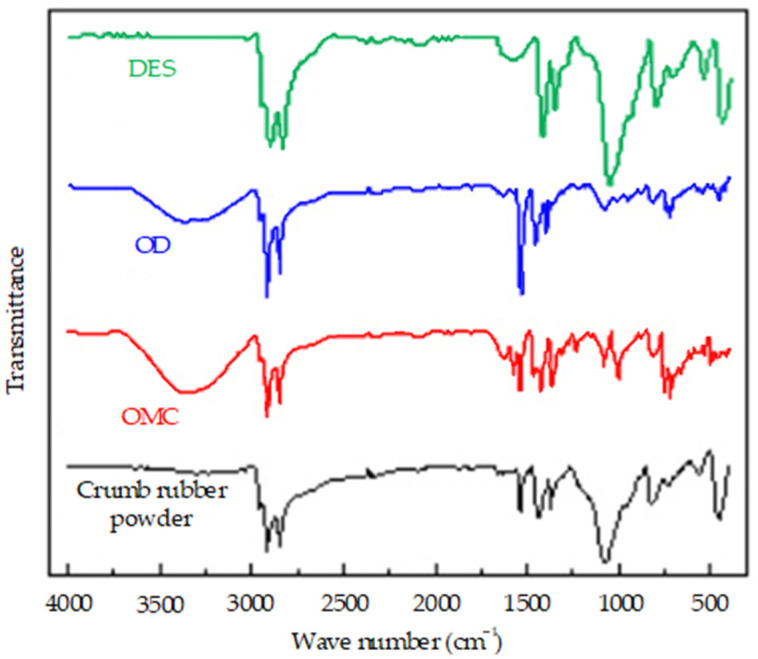
The infrared spectrum of the crumb rubber prepared by the chemical activation treatment method using different adjuvants.

The crumb rubber powder with 30 mesh was treated with different adjuvants including OMC (accounting for 3% of the crumb rubber powder by mass), OD (accounting for 3% of the crumb rubber powder by mass), and the self-made adjuvant, DES (accounting for 10% of the crumb rubber powder by mass). After that, the infrared spectrum test on the activated crumb rubber powder prepared by the chemical treatment method was carried out, and the test results of the activated crumb rubber powders are shown in Figure 8.

It can be seen from Figure 8 that both OMC and OD can break the polysulfide cross-linking bond at wave number 451 cm^−1^ in the crumb rubber powder and keep the “C=C” main chain in the crumb rubber powder intact. After the chemical treatment by OD, polar groups such as the hydroxyl groups formed on the surface of crumb rubber powder, and the surface activation of the crumb rubber powder was improved. At the same time, combined with the relevant phenomena in the test process, it was concluded that the solubility of OD and OMC in toluene is only medium, and these two adjuvants do not play all roles. Therefore, if a good solvent is used to increase the solubility of OD and OMC, the activation effect of the crumb rubber powder will be further promoted. In addition, it can be seen from Figure 8 that the adjuvant DES destroys the “C-S” bond in the crumb rubber powder to a great extent and achieves the effect of desulfurization. However, at the same time, it also destroys the “C=C” bond on the main chain of the crumb rubber powder molecules but does not damage other carbon chains, which achieves the effect of desulfurization activation.

#### 3.2.3. Activated Crumb Rubber Powder Prepared by the Mechanochemical Treatment Method

**Figure 9:**
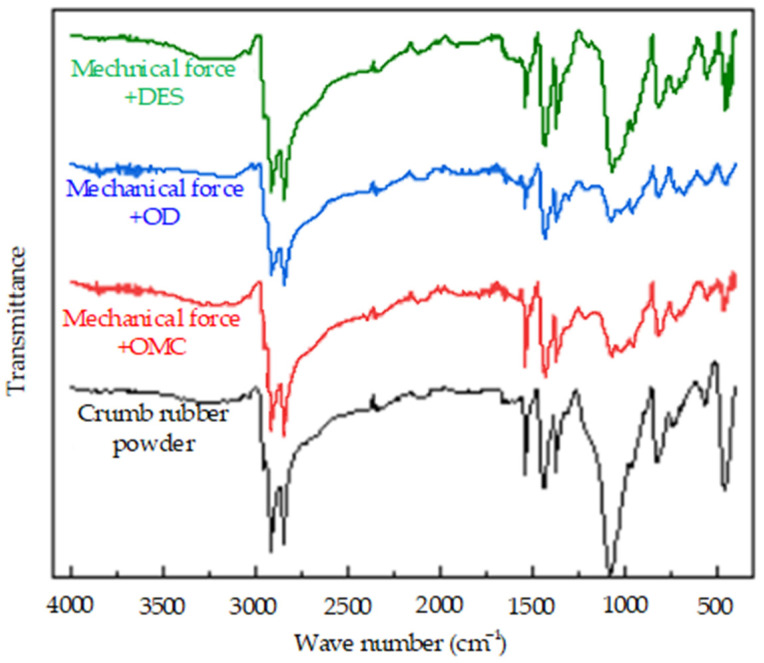
The infrared spectrum of the crumb rubber powder prepared by the mechanochemical activation treatment method using different adjuvants.

The crumb rubber powder with 30 mesh and OMC (accounting for 3% of the crumb rubber powder by mass), OD (accounting for 3% of the crumb rubber powder by mass), and the self-made adjuvant DES (accounting for 10% of the crumb rubber powder by mass) were mixed in the mixer at 120 °C for 60 min, and the infrared spectrum of the corresponding activated crumb rubber powder was tested. The test results of the activated crumb rubber powder are shown in Figure 9.

It can be seen from Figure 9 that the three adjuvants break the polysulfide cross-linking bond, such as that of “C-S” at the wave number of 451 cm^−1^, in the crumb rubber powder during the mixing process, and the strong absorption peak characterizing the “C=C” main chain at 1536 cm^−1^ was kept intact. The “C-S” bond was destroyed in the desulfurization process by the mechanochemical activation treatment method, and the “S=O” bond at 1082 cm^−1^ and the “C=O” bond at 1710 cm^−1^ were changed in the spectrum in varying degrees, as shown in Figure 9. OD and the self-made adjuvant DES also introduced polar groups such as “-OH” and “-COOH” on the surface of the crumb rubber powder, which would help to improve the surface activity of the crumb rubber powder.

**Figure 10:**
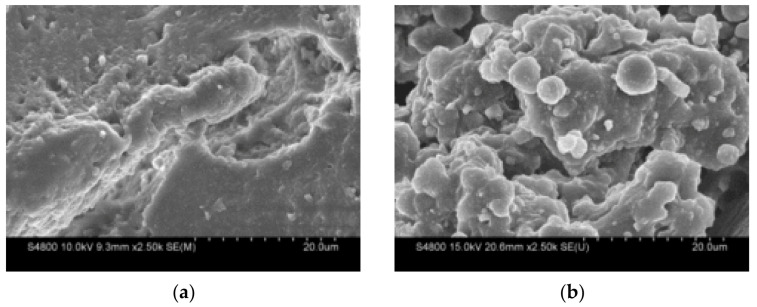

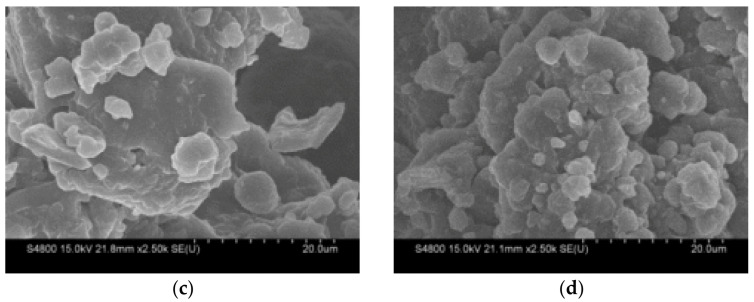
The infrared spectrum of the crumb rubber prepared by the heat activation treatment at different temperatures: (**a**) crumb rubber powder; (**b**) 160 °C + 60 min by heat treatment; (**c**) OMC by chemical treatment; and (**d**) mechanical force + OD by mechanochemical treatment.

This mechanochemical activation treatment usually changes the particle size distribution, and SEM of the crumb rubber particles before and after each treatment was provided, as shown in Figure 10. It can be seen that the surface of the crumb rubber powder after each activation treatment was rougher than that of the untreated crumb rubber powder. Obviously, the cross-linking structure on the surface and even inside of the crumb rubber powder after desulfurization and regeneration was damaged. The difference between different activation treatment methods lies in the gap distance and surface roughness (attached particles, debris, etc.) of the crumb rubber powder surface particles, which would influence the interfacial bonding between the crumb rubber powder and asphalt.

### 3.3. Performance Analysis of Activated Crumb-Rubber-Powder-Modified Asphalt Binder

Based on the existing literature [11], the asphalt binder modified by activated crumb rubber powder could be prepared, and according to the previous process exploration, the detailed steps were adopted to prepare the activated crumb-rubber-powder-modified asphalt binder.

#### 3.3.1. Asphalt Binder Containing the Activated Crumb Rubber Powder Prepared by the Heat Treatment Method

**Figure 11:**
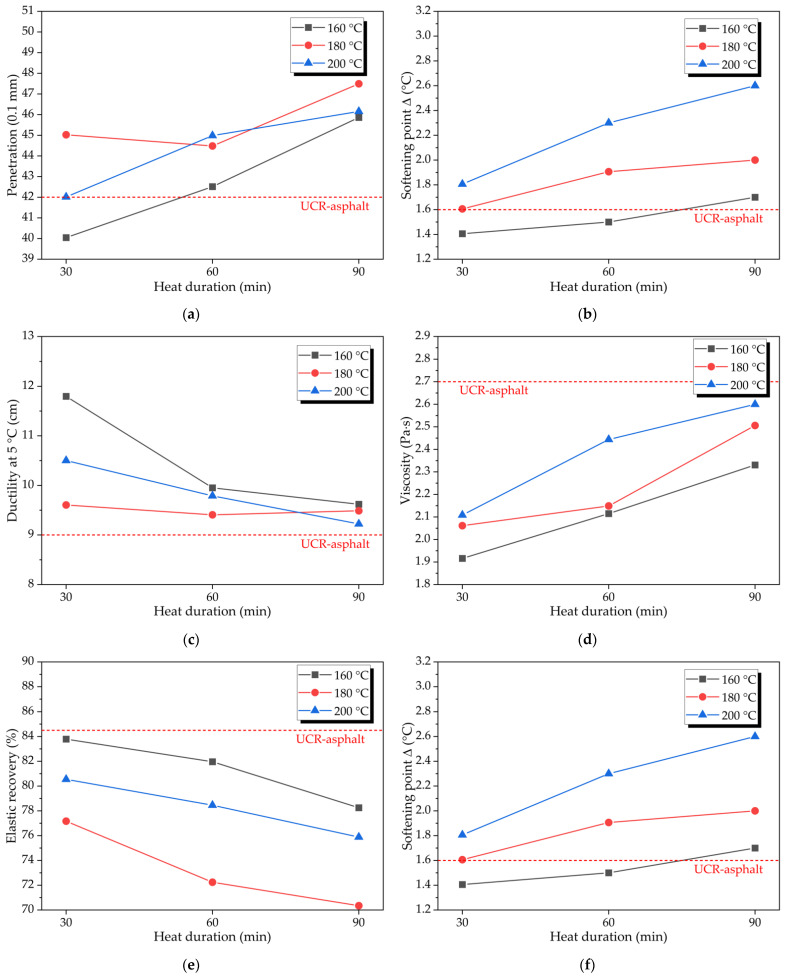
The conventional physical properties of asphalt binder containing activated crumb rubber powder prepared by the heat treatment method: (**a**) penetration; (**b**) softening point; (**c**) ductility at 5 °C; (**d**) viscosity; (**e**) elastic recovery; and (**f**) softening point Δ.

In this study, the crumb rubber powder with 30 mesh was activated by the heat treatment method at different temperatures (160 °C, 180 °C, and 200 °C) and for different durations (30 min, 60 min, and 90 min). Maoming 70# base asphalt was prepared by adding heat-treatment-activated crumb rubber powder (25% of base asphalt by mass) into base asphalt. According to the Chinese standard “Standard Test Methods of Bitumen and Bituminous Mixtures for Highway Engineering” (JTG E20-2011) and previous studies [30,31], the penetration, softening point, ductility at 5 °C, viscosity, elastic recovery, and softening point difference (Δ) were tested for the asphalt binder samples. According to the above experimental scheme, the prepared asphalt binder was tested to obtain the conventional physical properties, and the comparison results are shown in Figure 11.

It can be seen from Figure 11a,b that except for asphalt binder modified by the activated crumb rubber powder at the heat treatment temperature of 160 °C and the heat treatment duration of 30 min, the penetration values of the other activated crumb-rubber-powder-modified asphalt binders are higher than that of the untreated crumb-rubber-powder-modified asphalt binder (i.e., UCR asphalt binder). Moreover, the overall trend is that the higher the heat treatment temperature or the longer the heat treatment duration, the greater the penetration of the activated crumb-rubber-powder-modified asphalt binder. The softening point of the activated crumb-rubber-powder-modified asphalt binder gradually decreases with the increase in heat treatment duration and heat treatment temperature; Moreover, except for asphalt binder modified by the activated crumb rubber powder at the heat treatment temperature of 160 °C and for the heat treatment duration of 30 min, the softening point of the other activated crumb-rubber-powder-modified asphalt binders is lower than that of the UCR asphalt binder, and the high-temperature performance of the modified asphalt binder decreases after heat treatment. Therefore, the heat treatment method has a certain activation effect on the crumb rubber powder, and some crumb rubber restores the properties of raw rubber. Further, the softening point of the activated crumb-rubber-powder-modified asphalt binder decreases and the corresponding penetration increases.

As can be seen from Figure 11c,e, compared with the UCR asphalt binder, the ductility of the activated crumb-rubber-powder-modified asphalt binder increased. However, with the increase in heat treatment duration and heat treatment temperature, the ductility of the activated crumb-rubber-powder-modified asphalt binder decreased. After heat treatment of the crumb rubber powder, the elastic recovery rate of the modified asphalt binder decreased significantly. With the increase in heat treatment duration and heat treatment temperature, the elastic recovery rate of the activated crumb-rubber-powder-modified asphalt binder decreased and increased. Combined with the performance analysis results of the crumb rubber powder prepared by the heat treatment method, it can be known that the heat treatment method has a certain desulfurization activation effect on the crumb rubber powder, but when the heat treatment duration and temperature increase, the “C-C” main chain of the crumb rubber powder will be destroyed. Activated crumb rubber powder loses its strength and high elastic properties, and the ductility and elastic recovery rate of activated crumb-rubber-powder-modified asphalt binder decrease.

In Figure 11d,f, the 48 h segregation softening point difference (Δ) of the activated crumb-rubber-powder-modified asphalt binder increased with the increase in heat treatment duration and heat treatment temperature. The softening point difference Δ of most activated crumb-rubber-powder-modified asphalt binders is greater than that of the UCR asphalt binder. The viscosity of the activated crumb-rubber-powder-modified asphalt binder is lower than that of the UCR asphalt binder. However, with the increase in heat treatment duration and temperature, the viscosity of activated crumb-rubber-powder-modified asphalt binder increased. The reason analysis shows that the heat treatment method has a certain desulfurization activation effect on the crumb rubber powder. However, when the heat treatment duration and temperature are large, the “C-C” main chain of the crumb rubber powder will be destroyed and some rubber powder would be carbonized, which leads to the increase in softening point difference Δ and viscosity.

According to the above analysis, the heat treatment method has a certain activation effect on the crumb rubber powder. However, when the heat treatment duration is short and the heat treatment temperature is low, the desulfurization activation effect of heat treatment is not obvious. When the heat treatment duration is long and the heat treatment temperature is high, the “C-C” main chain in the crumb rubber powder will be interrupted and the crumb rubber powder will be partially carbonized, resulting in the decline of the properties of the activated crumb rubber powder and the activated crumb-rubber-powder-modified asphalt binder. The heat treatment method would not achieve the purpose of significantly reducing the viscosity and softening point Δ of the crumb-rubber-powder-modified asphalt binder.

#### 3.3.2. Asphalt Binder Containing Activated the Crumb Rubber Powder Prepared by the Chemical Treatment Method

**Figure 12:**
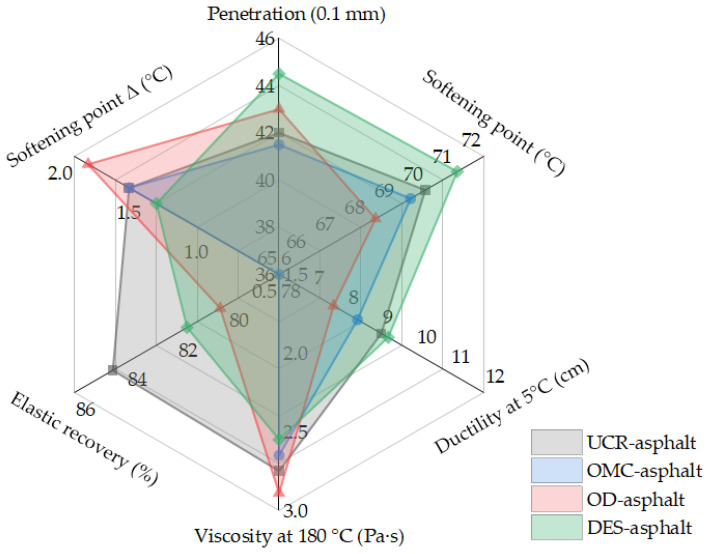
Radar chart for the chemical adjuvants based on the physical properties of the asphalt binder containing the activated crumb rubber powder prepared by the chemical treatment method.

Two kinds of commercial adjuvants, OMC and OD, as well as the self-made activation adjuvant DES, were selected for the activation treatment of the crumb rubber powder. The activated crumb rubber powder was prepared by mixing OMC (accounting for 3% of the crumb rubber powder by mass), OD (accounting for 3% of the crumb rubber powder by mass), and the self-made adjuvant DES (accounting for 10% of the crumb rubber powder by mass) with a certain amount of tthe crumb rubber powder with 30 mesh. After that, the activated crumb rubber powder (25% of the base asphalt by mass) was added into Maoming 70# base asphalt to prepare the activated crumb-rubber-powder-modified asphalt binder and test its performance. The conventional physical properties including the penetration, softening point, ductility at 5 °C, viscosity, elastic recovery, and softening point difference (Δ) of the activated crumb rubber powder prepared by the chemical treatment method are shown in Figure 12. According to the test results in Figure 12 and the performance analysis results of the activated crumb rubber powder prepared by the chemical treatment method, the crumb rubber powder has an obvious activation effect after chemical treatment by the different adjuvants. However, the viscosity and softening point difference Δ of the activated crumb-rubber-powder-modified asphalt binder are not observed to decrease significantly, which would not reflect the desulfurization activation effect of the crumb rubber powder. The analysis shows that there are residual reagents and water in the activated crumb rubber powder in the process of the chemical treatment. The existence of these impurities will affect the asphalt modification effect and reduce the performance of the activated crumb-rubber-powder-modified asphalt binder, which could not achieve the expected purpose.

#### 3.3.3. Asphalt Binder Containing the Activated Crumb Rubber Powder Prepared by the Mechanochemical Treatment Method

**Figure 13:**
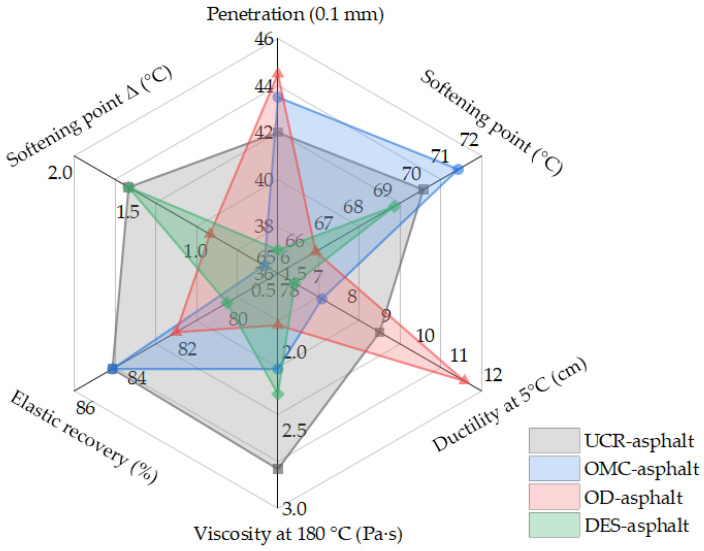
Radar chart for the chemical adjuvants based on the physical properties of the asphalt binder containing the activated crumb rubber powder prepared by the mechanochemical treatment method.

Referring to the process in the previous study [11], using the mechanochemical treatment method, the commercial adjuvants OMC and OD, as well as the self-made activation adjuvant DES, were selected for the activation treatment of the crumb rubber powder. The activated crumb rubber powder was prepared by mixing OMC (accounting for 3% of the crumb rubber powder by mass), OD (accounting for 3% of the crumb rubber powder by mass), and the self-made adjuvant DES (accounting for 10% of the crumb rubber powder by mass) with a certain amount of the crumb rubber powder with 30 mesh under the conditions of 60 minutes of mixing time and a 120 °C mixing temperature. Then, the activated crumb rubber powder (25% of the base asphalt by mass) was added into Maoming 70# base asphalt to prepare the activated crumb-rubber-powder-modified asphalt binder and test its performance. The conventional physical properties of the activated crumb rubber powder prepared by the mechanochemical treatment method are shown in Figure 13. It can be seen from Figure 13 that the viscosity values of the activated crumb-rubber-powder-modified asphalt binder obtained after being treated with OMC, OD, and DES are reduced compared with the UCR asphalt binder. When OD is used, the segregation softening point difference Δ of the activated crumb-rubber-powder-modified asphalt binder after storage for 48 h is also reduced, and the ductility is significantly improved. The purpose of improving the compatibility between the crumb rubber powder and asphalt and reducing the viscosity of the crumb-rubber-powder-modified asphalt binder is achieved.

### 3.4. Grey Relational Analysis

Grey relational analysis is a method to measure the relations between factors according to the similar or different procedures of the development trend for those factors [32,33,34]. By calculating the relational degree between the target values (reference sequence) and the influencing factors (comparison sequence), the relational degree is sorted to find the main factors affecting the target value. The analysis and comparison of these factors is essentially the analysis of geometric shapes between several curves, that is, the closer the geometric shape is, the closer the development and change trend is, and the greater the relational degree is.

The reference sequence is:

*X*_0_ = {*X*_0_(1), *X*_0_(2), …, *X*_0_(*n*)}, (1)

The comparison sequence is:

*X_i_* = {*X_i_*(1), *X_i_*(2),…, *X_i_*(*n*)}, *i* = 1, 2, …, *m* (2)

Each of the above sequences is averaged, that is, the ratio of each sequence to the average value is used, and then a new reference sequence *Y*_0_ and a new comparison sequence *Y_i_* can be obtained.

The relational coefficient between the comparison sequence and the reference sequence at time *k* (index and space) is:

(3) $$\xi_{i}(k)=\frac{\underset{i}{\min}\;\underset{k}{\min}\left|Y_{0}(k)-Y_{i}(k)\right|+\zeta \underset{i}{\max}\;\underset{k}{\max}\left|Y_{0}(k)-Y_{i}(k)\right|}{\left|Y_{0}(k)-Y_{i}(k)\right|+\zeta \underset{i}{\max}\;\underset{k}{\max}\left|Y_{0}(k)-Y_{i}(k)\right|},\;k=1,\;2,\;\ldots ,\;n$$

where *ζ* is the resolution coefficient in the internal (0, 1), and *ζ* is set as 0.5 in this study.

The relational degree is calculated as follows:

(4) $$\gamma_{i}=\frac{1}{n}\sum_{k=1}^{n}\xi_{i}(k)$$

where the order of the relational degree (*γ_i_*) determines the importance of the influencing factors. The larger the *γ_i_* value, the closer the development trend of *X_i_* and *X*_0_, and the greater the impact of *X_i_* on *X*_0_.

**Figure 14:**
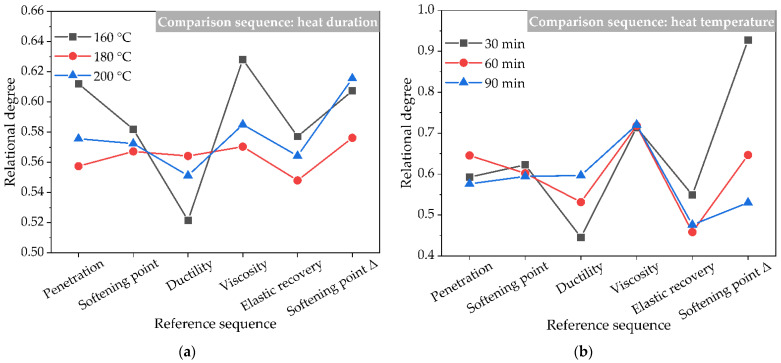

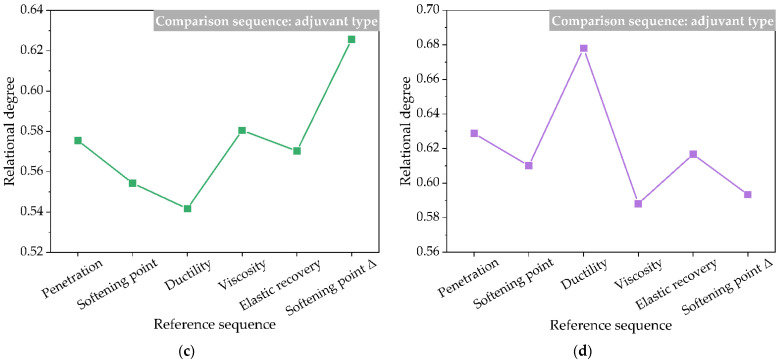
The grey relational degree results taking conventional physical properties as reference sequences considering different comparison sequences: (**a**) heat duration; (**b**) heat temperature; (**c**) adjuvant types for the chemical treatment; and (**d**) adjuvant types for the mechanochemical treatment.

The grey relational degree results taking conventional physical properties as reference sequences considering different comparison sequences are shown in Figure 14. In Figure 14a, generally speaking, the relational degree of the softening point difference Δ index representing the asphalt construction workability is the highest, followed by the relatively high relational degree of viscosity. The relational degree values of penetration and softening point are higher than those of ductility, indicating that the effect of heat treatment duration on the high-temperature stability of the asphalt binder is greater than that on the low-temperature performance. As shown in Figure 14b, the relational degree of the viscosity index of the asphalt binder is the highest, followed by the relational degree of the penetration and softening point difference Δ index, indicating that the heat treatment temperature has a great effect on the construction workability and high-temperature stability of the asphalt binder, while the relational degree values of ductility and elastic recovery are smaller. According to the relational degree results in Figure 14c, the adjuvant type for the chemical treatment has a greater effect on the construction workability and elastic recovery of the asphalt binder, while the effect of the adjuvant type for the chemical treatment on the high- and low-temperature performances of the asphalt binder is smaller. However, for the adjuvant type for the mechanochemical treatment, the relational degree of ductility representing the low-temperature performance of the asphalt binder is the highest. Therefore, based on the above grey relational degree analysis of the conventional physical properties of asphalt binder modified by crumb rubber powder under different activation modes, different activated crumb rubber powders can be selected and used to modify asphalt materials for different application requirements to obtain the corresponding modified asphalt binder.

## 4. Conclusions

The paper studied the influence of activation modes including heat treatment, chemical treatment, and mechanochemical treatment methods on crumb rubber powder and the corresponding rubber modified asphalt binder. According to the test results, the following conclusions can be drawn:

(1) The used crumb rubber sample with 30 mesh had a higher rubber hydrocarbon content and was suitable for asphalt modification. At the same time, the infrared spectroscopy and elemental analysis showed that the crumb rubber powder was mainly composed of alkanes, alkenes, sulfonic acids, aromatics, and a little silica rubber and antioxidant zinc oxide.

(2) The simple heat activation treatment method is not enough to greatly destroy the cross-linking structure of thhe crumb rubber powder, but the “C=C” bond was destroyed more seriously. Under the action of adjuvants in the chemical and mechanochemical treatments, the polysulfide cross-linking bond could be broken in the crumb rubber powder and the “C=C” main chain in the crumb rubber powder coiuld be kept intact.

(3) The performance improvement of the asphalt binder containing the activated crumb rubber powder after the heat and chemical treatments is not obvious, which does not achieve the purpose of reducing the viscosity and improving the compatibility of the rubber asphalt binder through desulfurization activation. The mechanochemical treatment would help to improve the performance of crumb-rubber-powder-modified asphalt binder.

(4) The data correlation analysis based on the grey relational degree can provide a reference for the selection of activated crumb rubber powders for different application requirements in the asphalt modification procedure.

In the future, other techniques will be adopted to analyze the influence of different activation treatments on crumb rubber and modified asphalt binder samples.

## Figures and Tables

**Table 1:** The sieved results of the crumb rubber sample with 30 mesh.

Sieves (Mesh)	20	30	40	60	80	100	120	200
30 mesh rubber (%)	100	97.3	56.8	26.3	20.0	5.9	0.4	0

**Table 2:** The composition results of the crumb rubber sample with 30 mesh.

VOCs (%)	TOM (%)	Acetone Extract (%)	Rubber Hydrocarbon (%)	Carbon Black (%)	Ash Content (%)
4.81	54.57	5.67	48.90	33.75	6.87

**Table 3:** The element content results of the crumb rubber sample with 30 mesh.

Sieves	Mass	N (%)	C (%)	H (%)	S (%)
30 mesh rubber	1.277	0.45	80.63	7.242	1.896

## Data Availability

Not applicable.
